# Understanding Ebola Virus Transmission

**DOI:** 10.3390/v7020511

**Published:** 2015-02-03

**Authors:** Seth Judson, Joseph Prescott, Vincent Munster

**Affiliations:** Laboratory of Virology, Division of Intramural Research, National Institute of Allergy and Infectious Diseases, National Institutes of Health, Hamilton, MT 59840, USA; E-Mails: sethdjudson@gmail.com (S.J.); prescottjb@niaid.nih.gov (J.P.)

**Keywords:** Ebola virus, transmission, epidemiology, experiments, airborne, fomite, droplet, environmental stability, BSL-4, filovirus

## Abstract

An unprecedented number of Ebola virus infections among healthcare workers and patients have raised questions about our understanding of Ebola virus transmission. Here, we explore different routes of Ebola virus transmission between people, summarizing the known epidemiological and experimental data. From this data, we expose important gaps in Ebola virus research pertinent to outbreak situations. We further propose experiments and methods of data collection that will enable scientists to fill these voids in our knowledge about the transmission of Ebola virus.

## 1. Introduction

The 2014 outbreak of Ebola virus disease (EVD) in West Africa has claimed more lives than all previous EVD outbreaks combined [1]. Along with its high case fatality rate, this outbreak has caused infection of several local and foreign health care workers. In order to understand outbreak control and determine appropriate public health practices, as well as guide future avenues of research, it is important to assess the current state of our knowledge about Ebola virus transmission between people (Table 1).

**Table 1:** Knowledge about different routes of Ebola virus transmission.

Mode of transmission	Consensus likelihood of occurring	Known	Unknown
Airborne/Aerosol (small droplet/droplet nuclei)	Unlikely from epidemiology of disease	EBOV can be aerosolized mechanically and cause lethal disease in non-human primates at low concentrations [2,3]	Ability of the virus to become airborne through respiratory tract in humans and animals
		Outbreaks contained without airborne precautions in the affected population [4]	Airborne stability of EBOV in tropical climates
		EBOV detected after 90 min in experimental small aerosols [5]	Whether AGPs produce EBOV aerosols that cause transmission
Fomite	Less likely from environmental sampling	Virus found in dried blood [6]	EBOV stability in tropical climates and on surfaces
		Persists on glass and in the dark for 5.9 days [7]	
Droplet (large droplet)	Likely from epidemiology and experiments	EBOV found in stool, semen, saliva, breast milk [6]	Whether infectious fluids are formed into droplets by humans
		Accidental infections in non-human primates, possibly from power washing [8,9]	
			Range of droplets containing EBOV
		EBOV infections without direct contact [10]	
Bodily fluids contact	Very likely from epidemiology and experimental data	Sharing needles and handling the deceased or sick are high risk factors [11]	How much virus is shed in different fluids
		EBOV found in a variety of bodily fluids [6]	

**Figure 1:**
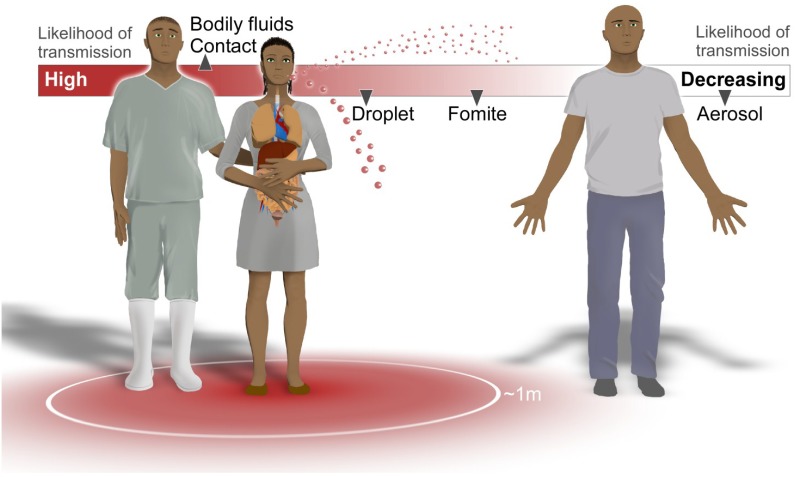
Potential routes of Ebola virus transmission and infection between people. Ebola virus (EBOV) has been isolated from bodily fluids including blood, stool, semen, saliva, and breast milk [6]; contact with these fluids from infected individuals creates a high risk of transmission. These infectious fluids can also be formed into droplets which travel in the air (range unknown, possibly 1 meter) and potentially infect others. EBOV has been detected in dried blood and persists on surfaces, so the possibility of fomite transmission exists. Airborne transmission via small aerosol droplets is unlikely from current EBOV epidemiology.

Ebola virus (EBOV), species *Zaire ebolavirus*, one of five species of viruses in the genus *Ebolavirus*, has been identified as the etiological agent of the 2014 outbreak of Ebola virus disease (EVD). In humans, EBOV has been found in variety of bodily fluids, including saliva, blood, breast milk, stool, and semen [6]. There are also multiple potential routes of transmission, including direct contact, fomite, droplet, and aerosol (Figure 1). Experiments involving non-human primates (NHPs) suggest EBOV can successfully infect after oral, conjunctival, respiratory, intramuscular, intraperitoneal and submucosal administration [2,12]. Infectious doses of less than 10 plaque-forming units (pfu) of EBOV have been reported to cause viremia in NHPs, depending on the route of administration [2]. Therefore, EBOV is highly infectious, for a low dose of virus is sufficient to cause disease, and EBOV is contagious; it is shed in multiple bodily secretions and easily transmitted through contact with these fluids.

Assessing the potential routes of EBOV transmission, the United States Centers for Disease Control and Prevention (CDC) has communicated the scientific consensus that EBOV spreads only through direct contact with mucous membranes or through broken skin with infected blood or bodily fluids, contaminated objects such as needles, and contact with infected animals [13]. Additionally, healthcare personnel are given precautions against droplet transmission.

Our knowledge about human-to-human EBOV transmission is based mainly on epidemiological evidence from previous outbreaks [14]. While authors have addressed what is known about EBOV transmission, many unanswered questions remain. Experiments should augment our knowledge about EBOV transmission and improve our current retrospective understanding. Therefore, we review the present knowledge regarding EBOV transmission and examine how research could answer remaining questions.

## 2. Potential Routes of EBOV Transmission

### 2.1. Airborne/Aerosol

Airborne or aerosol transmission of a virus occurs when small, virus-laden droplets evaporate before settling on surfaces, leaving behind infectious droplet nuclei that can travel long distances. The small droplets that can form these droplet nuclei are often called aerosols, but aerosols are also generally defined as any small liquid or solid particles that are suspended in air [15]. In order to better define biological aerosols, two categories have been created: small droplets and large droplets. The size cut-off for the diameter of small droplets and large droplets has been disputed, with some proposing small droplets to be <20 µm [15], and the size of droplet-nuclei to be <5 µm [16]. Here, we use the terms small droplet and aerosol interchangeably to describe particles that have the potential to form droplet-nuclei, and we use the phrases airborne and aerosol transmission synonymously to describe transmission via these particles.

Currently no data exist for whether EBOV forms droplet nuclei. EBOV particles have been found in human alveoli [17], yet it is not known if small droplets containing EBOV form within the human respiratory tract. However, epidemiological data have led to the understanding that EBOV does not undergo traditional airborne transmission. The majority of patients in previous epidemics have been infected by direct contact [10,11] and all EVD outbreaks in Africa have been contained without precautions against airborne transmission in the affected populations [4].

While there is no evidence of airborne transmission of EBOV in humans, experiments in NHPs have shed light on this mode of infection. Early experiments examining routes of infection of EBOV demonstrated that the virus could be aerosolized to small droplet or droplet nuclei size, and cause lethal disease in rhesus macaques after inhalation of at least 400 pfu [3]. More recent experiments have shown that inhalation of <10 pfu of EBOV is sufficient to cause lethal disease in NHPs [2]. However, it is important to note that in these experiments the virus was aerosolized mechanically, and they do not address the question of whether EBOV is aerosolized naturally. These studies were also done at temperate conditions, 24 degrees Celsius and <40% relative humidity, and therefore do not reflect the environment of African outbreaks. Temperate conditions were chosen to simulate an outbreak in the United States, and it has been shown that 24 degrees Celsius and <40% relative humidity increase the stability of other African hemorrhagic fever viruses, such as Lassa and Rift valley fever viruses [3].

Researchers have also tested the stability of mechanically generated aerosols (1–3 µm diameter) that contain EBOV. These stability studies have shown that 99% of the initial virus decays after 104 min at 50%–55% relative humidity and 19–25 degrees Celsius [5]. However, no one has tested the stability of EBOV in aerosols at the temperature or humidity that one would find in West Africa.

Initial concerns about the possibility of aerosol transmission of EBOV were raised when NHPs infected with another virus in the genus *Ebolavirus*, Reston virus, indirectly infected naïve NHPs in distant cages [3]. Indirect EBOV infections between infected and control NHPs and between oronasally infected pigs and uninfected NHPs caused further speculation about the airborne potential of EBOV [8,9]. However, as the authors note, husbandry practices, such as cleaning the cages of infected animals, could have contributed to forming droplets or aerosols containing infectious EBOV [9]. In comparison, EBOV transmission did not occur when intramuscularly infected NHPs were placed 0.3 m apart from uninfected NHPs separated by a plexiglass barrier [18].

Overall, differences in experimental design make it difficult to know what we can infer from these experiments about traditional airborne transmission. Measures such as the plexiglass barrier may prevent the exchange of droplets, but they may also impede aerosols. Furthermore, all EBOV transmission experiments take place in biosafety level 4 (BSL-4) laboratories that have pressure driven airflow, which could interfere with the path of the virus in the air. In order to definitely prove or disprove the possibility of EBOV airborne transmission in NHPs, rigorous experimental design is necessary to make sure droplet or fomite transmission cannot occur, while a positive control must also demonstrate that aerosol transmission is possible in the experimental setup.

### 2.2. Droplet

Droplet transmission, or large droplet transmission, refers to contact with infectious droplets that do not evaporate or travel long distances. Although these droplets do travel through the air, they are defined differently from aerosols that evaporate in the air to form droplet nuclei. While some viruses are not stable as droplet nuclei, they may remain stable inside droplets and can infect individuals exposed to them. It has been generally assumed that droplet transmission can occur up to a meter from an infected individual, but this varies depending on the droplet size, environment, and stability of the virus [19]. EBOV-containing droplets could form from multiple bodily fluids, including blood, saliva, vomit, and stool, yet no experimental studies have been done with EBOV droplets, so the range of transmission is not known. However, health care workers are given precautions against droplet transmission because of epidemiological evidence. In one outbreak, 5 out of 19 patients reported not having direct contact with an infected individual [10]. Because these patients were in close proximity with infected individuals, some speculate that droplet transmission could have occurred [10].

Other ways that EBOV-containing droplets and aerosols could be created are through aerosol-generating procedures (AGPs). Known AGPs in clinical settings are those that stimulate coughing or involve the manipulation of the patient’s airway, including activities such as intubation, manual ventilation, and broncoscopy. Although health care workers may not perform AGPs in ETUs, it is possible that those working in hospital settings could generate EBOV aerosols when using AGPs to treat an EVD patient.

Additional AGPs include spraying and pressure washing infected materials. For example, the previously mentioned indirect EBOV infections in NHPs are believed to have occurred from activities done by the researchers, such as pressure washing cages, or from activities by the NHPs themselves that could have formed EBOV-containing droplets that infected other animals. Although such AGPs might not occur outside of a laboratory setting, it would be helpful to experimentally determine the types of research and clinical activities that generate infectious EBOV droplets and aerosols. Air sampling while washing the cages of EBOV infected animals or when intubating an infected animal could reveal whether these activities generate EBOV droplets or aerosols. Additionally, this sampling could be performed during other experiments, thereby limiting the use of animals.

Experimentally testing the stability of the virus in bodily fluids such as vomit, saliva, stool, and blood could help us determine how long EBOV can persist in droplets generated from infected patients. Similar environmental stability experiments to those we described for small aerosols could also be done with large droplets to determine how factors such as temperature, humidity, and particle diameter influence the viability of EBOV. By determining the viability of the virus in different size aerosols and droplets, we could better predict the range and modes of EBOV transmission. Additionally, conducting air sampling in Ebola treatment units (ETUs) could reveal whether EBOV aerosols or droplets are generated by patients. These data could inform us whether personal protective equipment (PPE) guidelines and protective behaviors in ETUs are sufficient. For example, the CDC currently recommends that healthcare workers wear a minimum of a face shield, surgical mask, gown, and two pairs of gloves when working with EBOV patients and that a purifying respirator (PAPR) or N95 respirator be used during an AGP with an EVD patient [20]. However, if EBOV aerosols are generated by patients, then a face shield and surgical mask may not be sufficient. Furthermore, N95 respirators have a lower protection factor than PAPRs, therefore experimentally determining whether EBOV aerosols are produced by AGPs could help us reevaluate these recommendations.

### 2.3. Fomites and Environmental Stability

A fomite refers to any surface that a pathogen is able to persist on, and fomite transmission can occur when an individual comes into contact with that infected surface. Potential routes of EBOV fomite transmission include touching objects such as bed sheets or gurneys that have been in contact with EBOV patients or other surfaces contaminated with EBOV-containing bodily fluids.

Little is known about the stability of EBOV on surfaces. One experiment showed that EBOV viral load is reduced by 4 log_10_ after 5.9 days when placed on glass and in the dark at 24 °C and 40% relative humidity [7]. Another experiment showed that EBOV could be recovered after 50 days, when dried in culture media on glass at 4 °C [5]. Limited environmental testing in outbreak locations has shown little evidence for EBOV persistance on surfaces. An assessment for the presence of EBOV in an African hospital found only 2 of 33 samples from surfaces inside and outside the hospital ward to be RT-PCR positive, while virus was present in 16 of 54 clincal samples [6]. Both positive environmental samples contained dried blood. Routine cleaning of the hospital wards could have eliminated EBOV on hospital surfaces, or the virus might not be stable in this environment.

Further experiments testing the stability of EBOV on surfaces found in outbreak locations and in tropical conditions could determine whether EBOV persists in the environment and if it is possible to acquire infection through contact with contaminated surfaces. In these laboratory experiments it is also important to accurately mimic the conditions one would find in real outbreak settings. While previous studies tested the stability of EBOV in dried guinea pig sera and cell culture media at low temperature and humidity, it would be insightful to test the stability of EBOV in dried human bodily fluids and at a higher temperature and humidity.

In addition to experimentally testing the stability and viability of EBOV in human bodily fluids, both as liquid and dried on different surfaces, it would be useful to test the limits of EBOV stability in other environments, such as water. Because of the reduced public health infrastructure in countries afflicted by EVD outbreaks, it is common to find open sewage canals or waste-containing buckets. Because EBOV is shed in feces and other bodily secretions that come into contact with water, it would be helpful to know how long the virus is stable in water. Contaminated water could come into contact with the eyes, nose, or mouth of individuals, leading to possible infection through contact with mucous membranes.

Other viruses that are also shed in vomit and diarrhea can cause infection among people who come into contact with sewage contaminated water [21]. Many of these viruses are non-enveloped enteric viruses, which are very stable in water [22]. In contrast, EBOV is a single-stranded RNA virus with a lipid envelope derived from its host cell [23]. Although enveloped viruses are generally less stable in water, it has been shown that enveloped RNA viruses can persist in water [22]. One enveloped RNA virus, bovine diarrhea virus, a member of the family *Flaviviridae*, persists for 19–30 days in water [21]. Yet HIV-1, also an enveloped RNA virus, only remains infectious in water for up to 96 h when it is cell-associated [24]. In fact, it is difficult to generalize about the stability of viruses in water and waste. Factors such as the species of virus, water content, waste type, and temperature all influence the environmental stability of an individual virus [22]. Therefore, the only way to make a conclusion about the stability of EBOV or other filoviruses in water and waste is to experimentally test them.

Many biological factors could influence the stability of EBOV, such as whether the virus is cell-associated or protected by organic matter such as fecal waste. Because there are so many permutations for testing the virus in different environments and at varying temperatures, humidities, pH’s, and salinities, it is most reasonable to test the virus in conditions that one is likely to find in outbreak settings. Designing experiments that accurately simulate the environments where one might encounter EBOV, such as on clinical surfaces, corpses, and disposed waste, and are at the same temperature and humidity as outbreak locations, would provide the environmental boundaries for EBOV transmission.

### 2.4. Contact with Bodily Fluids

Of all the possible modes of transmission, direct contact with bodily fluids remains the most likely way of transmitting EBOV between people. Circumstantial evidence from previous outbreaks, epidemiological data, and experiments in NHPs all demonstrate that contact with EBOV infected fluids via mucous membranes, injection, or open wounds can lead to infection.

The use of unsterilized needles was linked to the spread of EBOV in hospital wards during the first recorded EVD outbreak in the Democratic Republic of the Congo in 1976 [25]. Accidental exposures to Ebola virus have also occurred, with needlestick injuries leading to infections among researchers [26]. Contact with bodily fluids has also been implicated as the reason why caregivers often become infected after contact with patients. In a study of the risk factors associated with contracting Ebola virus during an outbreak in Kikwit, contact with bodily fluids strongly predicted risk of infection as did sharing hospital beds [11].

Despite contact with bodily fluids being a confirmed route of EBOV transmission, it is not clear when and which fluids contain infectious virus. EBOV has been isolated from blood, saliva, breast milk and semen [6], while RNA has been detected in sweat, stool, tears, and on skin, vaginal, and rectal swabs [14]. Although this gives us a broad understanding of the range of bodily fluids that may contain EBOV, we still do not know how much virus is secreted in these substances. Isolating EBOV from infected patients and determining the viable concentration of infectious virus during different time points of disease could elucidate how much virus is shed in different bodily fluids and when it is shed. However, it is challenging to collect these data during an outbreak and conduct retrospective laboratory analysis. Alternative solutions could be to sample bodily fluids from infected NHPs routinely post infection. While NHPs have been monitored during phases of EVD, one has yet to isolate and titrate virus from different secretions throughout the entire stages of the disease to determine whether viable virus is present. Equating RNA copy number to titrations of virus is currently the best clinical option for determining the amount of virus in different bodily fluids from infected patients. This has been done with another ebolavirus, Sudan virus, in sera, allowing researchers to compare the viral load in samples from patients who died and those that recovered [27]. Therefore, this needs to also be done with EBOV in samples from patients in the current epidemic.

One limitation of sampling the viral load in patients is that we may be missing asymptomatic or subclinical infected individuals. These asymptomatic individuals may have been recently infected and are in the pre-clinical stages of EVD, or they may have been infected for a while and have not developed any symptoms, remaining subclinical. EBOV replication has been identified in patients who never developed any symptoms after being exposed to the virus [28], but it is unknown whether these individuals may also secrete virus. Sudan virus RNA was typically found at low levels during the onset of symptoms [27], so it likely that asymptomatic, pre-clinical individuals have a low level of viremia. While EBOV transmission from these asymptomatic individuals seems very unlikely from epidemiological evidence, monitoring the viral load in both pre-clinical patients and those that have remained subclinical could definitively prove whether those who are asymptomatic are not contagious. However, the only way of identifying asymptomatic individuals is through testing those who have been exposed to EBOV but have not shown any symptoms, which is logistically challenging. Therefore, healthcare workers and family members who have been exposed to infected patients but have not developed symptoms would be the ideal target populations for these studies.

## 3. Why Experiments Are Necessary

While evidence from previous outbreaks has enabled public health personnel to make informed decisions during the current outbreak, we do not have a complete understanding of all of the potential routes of EBOV transmission. If we continue to piece together our understanding of transmission from primarily epidemiological data, we will miss understanding the biological phenomena that lead to transmission events.

Fortunately, BSL-4 research facilities are ideally equipped to help answer these underlying questions about EBOV environmental stability and transmission. Experiments testing different modes of transmission in animal models and the limits of EBOV viability in different environments could answer many of the remaining questions about EBOV transmission, such as whether the virus can undergo droplet or fomite transmission.

As we consider the current EBOV epidemic in regards to potential outbreaks of other viral diseases, it is important to think about how both epidemiology and experimental research can be used together to inform our understanding of virus transmission. Epidemiology allows us to infer how transmission is occurring on a population level, while experiments enable researchers to determine the biological limits of transmission. Therefore, the experiments that we propose for EBOV could also be used to fill gaps in our knowledge about other viruses as well.

In summary, sparse epidemiological and experimental evidence exist for mechanisms of EBOV transmission between people. Currently we know that humans shed the virus in a variety of bodily fluids, are infected by multiple routes, and only need a small amount of virus for infection. This creates a high risk of exposure, so all infection control measures must be followed rigorously in order to prevent further infections. From epidemiological evidence it appears that these infections primarily occur through direct contact with bodily fluids, yet more indirect modes of EBOV transmission have yet to be thoroughly studied experimentally. While contact with bodily fluids from EVD patients remains the most likely route of transmission, further experiments regarding the generation of EBOV in the respiratory tract and during AGPs, as well as the stability of EBOV in droplets and on surfaces, could help us define all routes of infection and improve infection control policies.

## References

[1] Centers for Disease Control and Prevention Outbreaks Chronology: Ebola Virus Disease. http://www.cdc.gov/vhf/ebola/outbreaks/history/chronology.html.

[2] Reed D.S., Lackemeyer M.G., Garza N.L., Sullivan L.J., Nichols D.K. (2011). Aerosol exposure to Zaire ebolavirus in three nonhuman primate species: Differences in disease course and clinical pathology. Microbes Infect..

[3] Johnson E., Jaax N., White J., Jahrling P. (1995). Lethal experimental infections of rhesus monkeys by aerosolized Ebola virus. Int. J Exp. Pathol..

[4] Borio L., Inglesby T., Peters C.J., Schmaljohn A.L., Hughes J., Jahrling P., Ksiazek T., Johnson K., Meyerhoff A., O’Toole T. (2002). Hemorrhagic fever viruses as biological weapons: Medical and public health management. JAMA.

[5] Piercy T., Smither S., Steward J., Eastaugh L., Lever M. (2010). The survival of filoviruses in liquids, on solid substrates and in a dynamic aerosol. J. Appl. Microbiol..

[6] Bausch D.G., Towner J.S., Dowell S.F., Kaducu F., Lukwiya M., Sanchez A., Nichol S.T., Ksiazek T.G., Rollin P.E. (2007). Assessment of the risk of Ebola virus transmission from bodily fluids and fomites. J. Infect. Dis..

[7] Sagripanti J.L., Rom A.M., Holland L.E. (2010). Persistence in darkness of virulent alphaviruses, Ebola virus, and Lassa virus deposited on solid surfaces. Arch. Virol..

[8] Jaax N., Jahrling P., Geisbert T., Geisbert J., Steele K., McKee K., Nagley D., Johnson E., Jaax G., Peters C. (1995). Transmission of Ebola virus (Zaire strain) to uninfected control monkeys in a biocontainment laboratory. Lancet.

[9] Weingartl H.M., Embury-Hyatt C., Nfon C., Leung A., Smith G., Kobinger G. (2012). Transmission of Ebola virus from pigs to non-human primates. Sci. Rep..

[10] Roels T., Bloom A., Buffington J., Muhungu G., Mac Kenzie W., Khan A., Ndambi R., Noah D., Rolka H., Peters C. (1999). Ebola hemorrhagic fever, Kikwit, Democratic Republic of the Congo, 1995: Risk factors for patients without a reported exposure. J. Infect. Dis..

[11] Dowell S.F., Mukunu R., Ksiazek T.G., Khan A.S., Rollin P.E., Peters C. (1999). Transmission of Ebola hemorrhagic fever: A study of risk factors in family members, Kikwit, Democratic Republic of the Congo, 1995. J. Infect. Dis..

[12] Jaax N.K., Davis K., Geisbert T.J., Vogel P., Jaax G.P., Topper M., Jahrling P.B. (1996). Lethal experimental infection of rhesus monkeys with Ebola-Zaire (Mayinga) virus by the oral and conjunctival route of exposure. Arch. Pathol. Lab. Med..

[13] Centers for Disease Control and Prevention Transmission: Ebola Virus Disease. http://www.cdc.gov/vhf/ebola/transmission/.

[14] Centers for Disease Control and Prevention Review of Human-to-Human Transmission of Ebola Virus: Ebola (Ebola Virus Disease). http://www.cdc.gov/vhf/ebola/transmission/human-transmission.html.

[15] Tellier R. (2009). Aerosol transmission of influenza A virus: A review of new studies. J. R. Soc. Interface.

[16] Tang J.W., Li Y., Eames I., Chan P.K., Ridgway G.L. (2006). Factors involved in the aerosol transmission of infection and control of ventilation in healthcare premises. J. Hosp. Infect..

[17] Martines R.B., Ng D.L., Greer P.W., Rollin P.E., Zaki S.R. (2015). Tissue and cellular tropism, pathology and pathogenesis of Ebola and Marburg viruses. J. Pathol..

[18] Alimonti J., Leung A., Jones S., Gren J., Qiu X., Fernando L., Balcewich B., Wong G., Stroher U., Grolla A. (2014). Evaluation of transmission risks associated with *in vivo* replication of several high containment pathogens in a biosafety level 4 laboratory. Sci. Rep..

[19] Johnson G.R., Morawska L., Ristovski Z.D., Hargreaves M., Mengersen K., Chao C.Y.H., Wan M.P., Li Y., Xie X., Katoshevski D. (2011). Modality of human expired aerosol size distributions. J. Aerosol. Sci..

[20] Centers for Disease Control and Prevention Guidance on Personal Protective Equipment To Be Used by Healthcare Workers during Management of Patients with Ebola Virus Disease in U.S. Hospitals, Including Procedures for Putting On (Donning) and Removing (Doffing): Ebola (Ebola Virus Disease). http://www.cdc.gov/vhf/ebola/healthcare-us/ppe/guidance.html.

[21] Bosch A. (2010). Human enteric viruses in the water environment: A minireview. Int. Microbiol..

[22] Sobsey M.D., Meschke J.S. (2003). Virus Survival in the Environment with Special Attention to Survival in Sewage Droplets and Other Environmental Media of Fecal or Respiratory Origin.

[23] Kawaoka Y. (2005). How Ebola virus infects cells. N. Engl. J. Med..

[24] Casson L.W., Ritter M.O., Cossentino L.M., Gupta P. (1997). Survival and recovery of seeded HIV in water and wastewater. Water Environ. Res..

[25] WHO/International Study Team (1978). Ebola haemorrhagic fever in Zaire, 1976. Bull. World Health Organ..

[26] Tarantola A., Abiteboul D., Rachline A. (2006). Infection risks following accidental exposure to blood or body fluids in health care workers: A review of pathogens transmitted in published cases. Am. J. Infect. Control..

[27] Towner J.S., Rollin P.E., Bausch D.G., Sanchez A., Crary S.M., Vincent M., Lee W.F., Spiropoulou C.F., Ksiazek T.G., Lukwiya M. (2004). Rapid diagnosis of Ebola hemorrhagic fever by reverse transcription-PCR in an outbreak setting and assessment of patient viral load as a predictor of outcome. J. Virol..

[28] Leroy E., Baize S., Volchkov V., Fisher-Hoch S., Georges-Courbot M., Lansoud-Soukate J., Capron M., Debre P., McCormick J., Georges A. (2000). Human asymptomatic Ebola infection and strong inflammatory response. Lancet.

